# Supplementary material for: Dietary Intervention with Cottonseed and Olive Oil Differentially Affect the Circulating Lipidome and Immunoregulatory Compounds—A Randomized Clinical Trial
Source: Metabolites. 2025 Sep 8;15(9):599. doi: 10.3390/metabo15090599 (PMC12472154; doi:10.3390/metabo15090599)
Supplement: Supplementary file 1 [file metabolites-15-00599-s001.zip › 20250805_metabolites-3783079_Supplementary_Revisions_final.pdf]

Supplementary Material

Title: Dietary intervention of cottonseed and olive oil differentially affect the circulating lipidome and immunoregulatory compounds – a randomized clinical trial

Authors: Gwendolyn Cooper[1], Prabina Bhattarai[2], Brett Sather[1], Marguerite L. Bailey[1], Morgan L. Chamberlin[2], Mary P. Miles[2, $], & Brian Bothner[2,$]

Affiliations:

[1] Department of Chemistry and Biochemistry, Montana State University, Bozeman, MT

[2] Department of Food Systems, Nutrition, and Kinesiology, Montana State University, Bozeman, MT

$ Correspondence: mmiles@montana.edu and bbothner@montana.edu

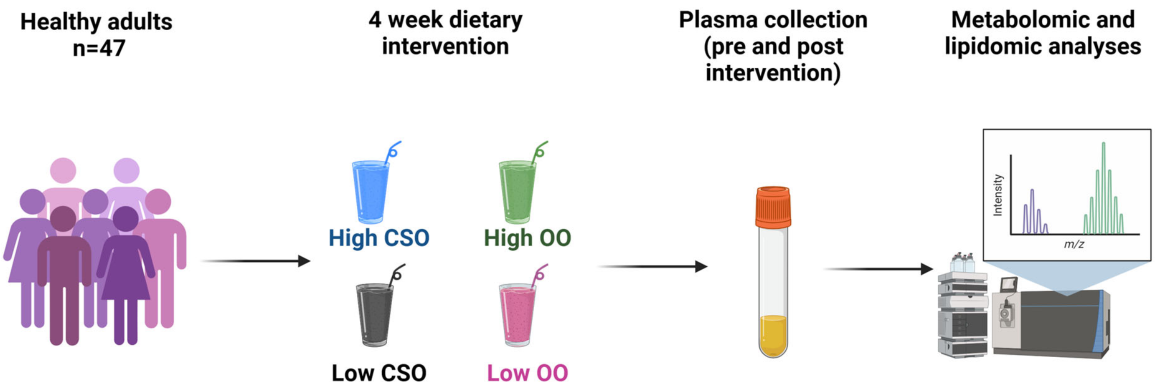

Figure S1: Double-blind, parallel study design overview.

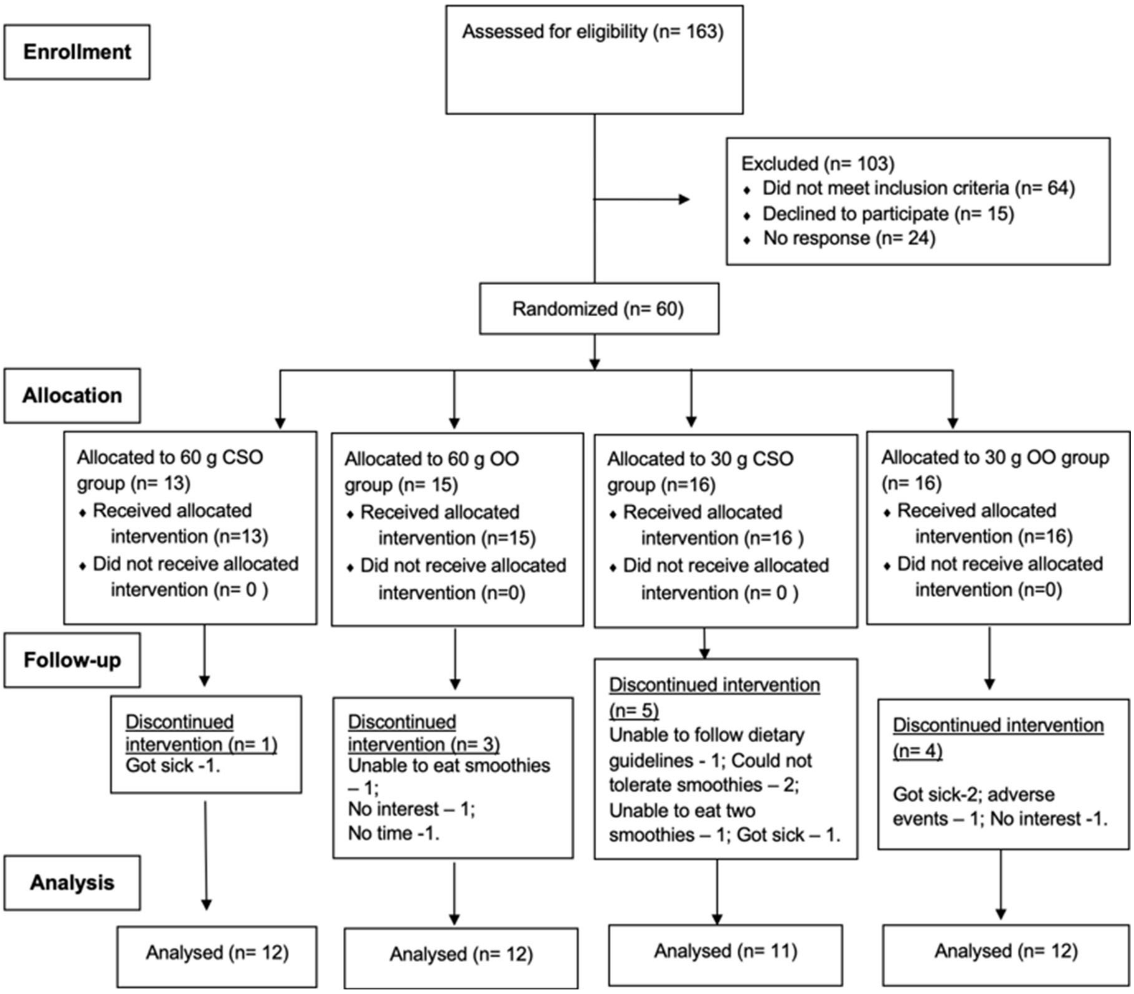

Figure S2: CONSORT flowchart

Smoothies	30 g CSO Mango	30 g CSO Chocolate	30 g OO Mango	30 g OO Chocolate	60 g CSO Mango	60 g CSO Chocolate	60 g OO Mango	60 g OO Chocolate
Energy (Kcal)	371.58	414.05	369.18	411.65	377.18	403.28	372.38	398.48
Fat (g)	16	16.9	16	16.90	31	31.4	31	31.4
Protein (g)	1.91	3.81	1.91	3.81	1.72	2.66	1.72	2.66
Carbohydrates(g)	59.53	68.61	59.53	68.61	25.22	30.52	25.22	30.52
Dietary fiber (g)	5.30	5.12	5.29	5.12	2.82	3.59	2.82	3.59
Calcium (mg)	114.24	104.04	114.24	104.04	110.85	99.79	110.85	99.79
Potassium (mg)	389.53	609.67	389.53	609.67	353.61	434.33	353.61	434.33
Thiamine (mg)	0.07	0.11	0.07	0.11	0.06	0.082	0.067	0.082
Riboflavin (mg)	0.10	0.19	0.1	0.19	0.08	0.13	0.08	0.13
Alpha-carotene (µg)	0	10.43	0	10.43	0	7	0	7
Beta - carotene (µg)	992	11.92	992	11.92	934.4	8	934.40	8
Saturated fat (16:0) (g)	3.41	3.41	1.82	1.82	6.81	6.81	3.63	3.63
MUFA (g)	2.55	2.55	10.17	10.17	5.34	5.34	20.76	20.76
MUFA	2.55	2.55	10.17	10.17	5.10	5.10	20.34	20.34

(18:1) (g)								
PUFA (18:2) (g)	7.73	7.73	1.26	1.26	15.45	15.45	2.52	2.52
PUFA (g)	7.79	7.79	1.36	1.36	15.57	15.57	2.72	2.72
ALA (g)	0.03	0.03	0.098	0.098	0.06	0.06	0.19	0.19
Alpha-tocopherol (g)	5.29	5.29	0	0	10.59	10.59	0	0
Total phytosterols (g)	47.25	47.25	27.79	27.79	94.5	94.5	55.58	55.58
N6:N3	257.50	257.50	12.90	12.90	257.50	257.50	12.90	12.90

Table S1: Smoothie nutritional composition

	Cholesterol (mmol/L)	HDL (mmol/L)	LDL (mmol/L)	VLDL (mmol/L)	IL_1b (pg/mL)	IL 10 (pg/mL)	IL_1ra (pg/mL)	IFNg (pg/mL)	TNF (pg/mL)	IL6 (pg/mL)	IL8 (pg/mL)
All (n=47)	4.19 ± 0.85	1.56 ± 0.38	2.22 ± 0.78	0.41 ± 0.13	1.72 ± 2.91	5.81 ± 5.27	109.49 ± 64.91	1.45 ± 1.73	7.70 ± 4.66	3.20 ± 3.31	3.66 ± 1.73
Female (n=23)	4.00 ± 0.75	1.67 ± 0.37	1.95 ± 0.58	0.38 ± 0.092	1.42 ± 2.72	5.13 ± 4.38	115.34 ± 73.28	1.30 ± 1.96	6.91 ± 2.15	3.00 ± 3.49	3.76 ± 2.11
Male (n=24)	4.37 ± 0.90	1.45 ± 0.37	2.48 ± 0.85	0.44 ± 5.87	2.00 ± 3.07	6.46 ± 5.93	103.89 ± 55.14	1.59 ± 1.46	8.46 ± 6.08	3.38 ± 3.11	3.56 ± 1.25

Table S2: Participant cholesterol and cytokine panels

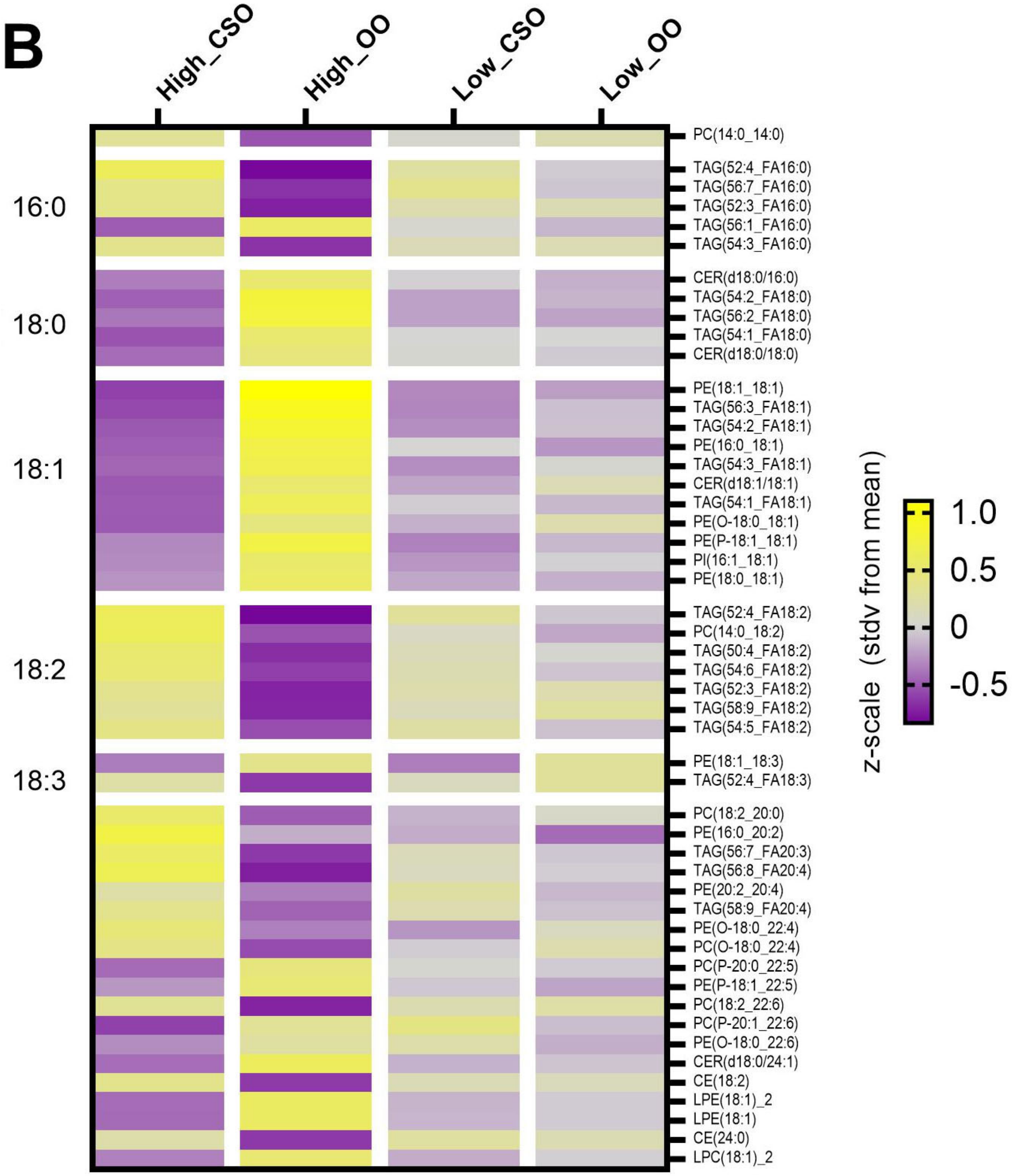

Figure S3: Zoomed in version of Figure 2B

A

B

C

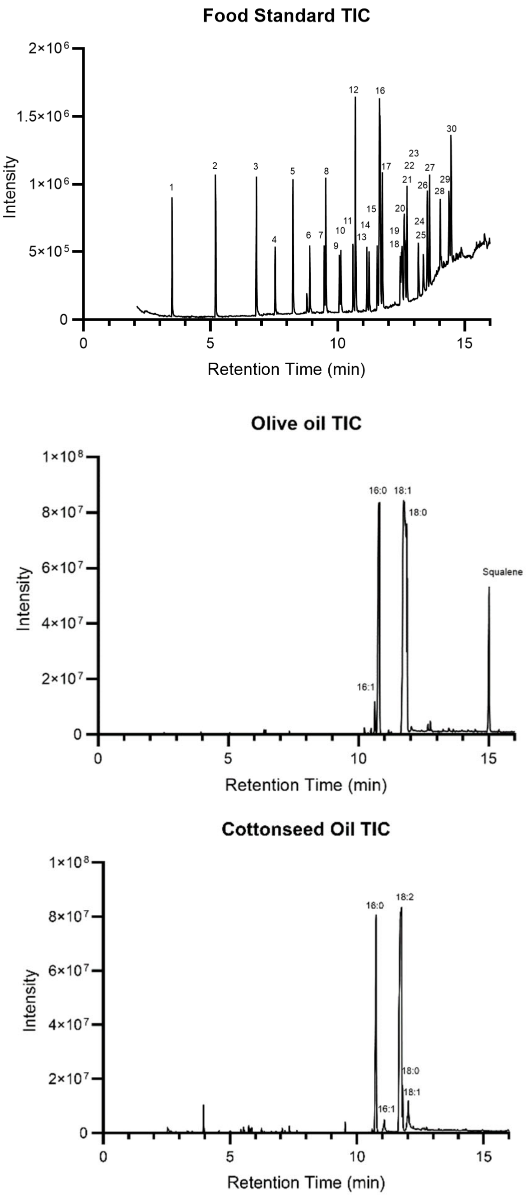

D

Number	RT (min)	Compound
1	3.4856	C6:0
2	5.194	C8:0
3	6.8012	C10:0
4	7.5402	C11:0
5	8.241	C12:0
6	8.9025	C13:0
7	9.472	C14:1
8	9.5307	C14:0
9	10.0748	C18:1
10	10.1285	C15:0
11	10.6015	C16:1
12	10.6991	C16:0
13	11.1497	C17:1
14	11.239	C17:0
15	11.5578	C18:3
16	11.6515	C18:2
17	11.7574	C18:0
18	12.4708	C20:4
19	12.5123	C22:5
20	12.5398	C18:3
21	12.6253	C20:2
22	12.6567	C20:3
23	12.7274	C20:0
24	13.181	C21:0
25	13.373	C22:6
26	13.5316	C22:1
27	13.6196	C22:0
28	14.0401	C23:0
29	14.3846	C24:1
30	14.4652	C24:0

Figure S4: GC-MS data from CSO, OO, and food standard. (**A**) F0ood standard total ion chromatogram (TIC). (**B**) Olive oil TIC. (**C**) Cottonseed oil TIC. (**D**) The fatty acids that correspond to the food standard peaks.

Name	Enzyme	PUFA	Class
15-deoxy-PGJ2	COX	AA	PG
PGF2a	COX	AA	PG
5,15-DiHEPE	ALOX5	EPA	DiHEPE
5,15-diHETE	ALOX5	AA	diHETE
5S,6R-diHETE	ALOX5	AA	diHETE
17,18 DiHETE	ALOX5	AA	diHETE
7,8-DiHDPA	CYP450	DHA	DiHDPA
13,14-DiHDPA	CYP450	DHA	DiHDPA
16,17-DiHDPA	CYP450	DHA	DiHDPA
19,20-DiHDPA	CYP450	DHA	DiHDPA
7,8-DiHDT	CYP450	DTA	DiHDTr
13,14-DiHDT	CYP450	DTA	DiHDTr
19,20-DiHDTA	CYP450	DPA	DiHDTA
9,10 DiHOME	CYP450	Linoleic	DiHOME
12,13 DiHOME	CYP450	Linoleic	DiHOME
5,6 EET	CYP450	AA	EET
10,11-EpDPA	CYP450	DPA	EpDPA
13,14-EpDPA	CYP450	DPA	EpDPA
16,17-EpDPA	CYP450	DPA	EpDPA
19,20-EpDPA	CYP450	DPA	EpDPA
11,12-EpETE	CYP450	EPA	EpETE
14,15-EpETE	CYP450	EPA	EpETE
8,9-EpETE	CYP450	EPA	EpETE
12(13)-EpOME	CYP450	Linoleic	EpOME
9(10)-EpOME	CYP450	Linoleic	EpOME
7(8)-EpDTA	CYP450	DPA	EpDTA
DHA	FA	DHA	DHA
AA	FA	AA	FA
DTA	FA	DTA	FA
DPA	FA	DPA	FA
EPA	FA	EPA	FA
ALA	FA	Linolenic	FA
LA	FA	Linoleic	FA
14-HDHA	ALOX12	DHA	HDHA
16-HDHA	CYP450	DHA	HDHA
4-HDHA	ALOX5	DHA	HDHA
14-HDTA	ALOX15	DTA	HDTA
16-HDTA	CYP450	DTA	HDTA
12-HEPE	ALOX12	EPA	HEPE
18-HEPE	CYP450	EPA	HEPE
5-HEPE	ALOX5	EPA	HEPE
11-HETE	COX	AA	HETE
12-HETE	ALOX12	AA	HETE
15-HETE	ALOX15	AA	HETE
20-HETE	CYP450	AA	HETE
5-HETE	ALOX5	AA	HETE
9-HETE	CYP450	AA	HETE
13-HODE	ALOX15	Linoleic	HODE
9-HODE	ALOX12	Linoleic	HODE
13(S)HOTrE	ALOX15	Linoleic	HOTrE
9(S)HOTrE	ALOX12	Linoleic	HOTrE
13-OxoODE (13-KODE)	ALOX15	Linoleic	OxoODE
20-OH-LTB4	ALOX5	AA	LTB4
15(R)-LXA4	Ox	AA	LXA4
RvD2	ALOX5	DHA	RvD
PD1	ALOX15	DHA	PD1
11-beta-PGF2a	COX	AA	PG
13,14-dihydro-15-keto PGD	COX	AA	PG
13,14-dihydro-15-keto PGF	COX	AA	PG
15-keto PGF2a	COX	AA	PG
20-OH PGF2a	COX	AA	PG
8-iso-PGF2a	COX	AA	PG
PGD2	COX	AA	PG
PGJ2	COX	AA	PG
TXB2	COX	AA	PG

Table S3: All LMs detected in participant samples. Enzyme, parent PUFA, and molecular class are also included for each detected LM.

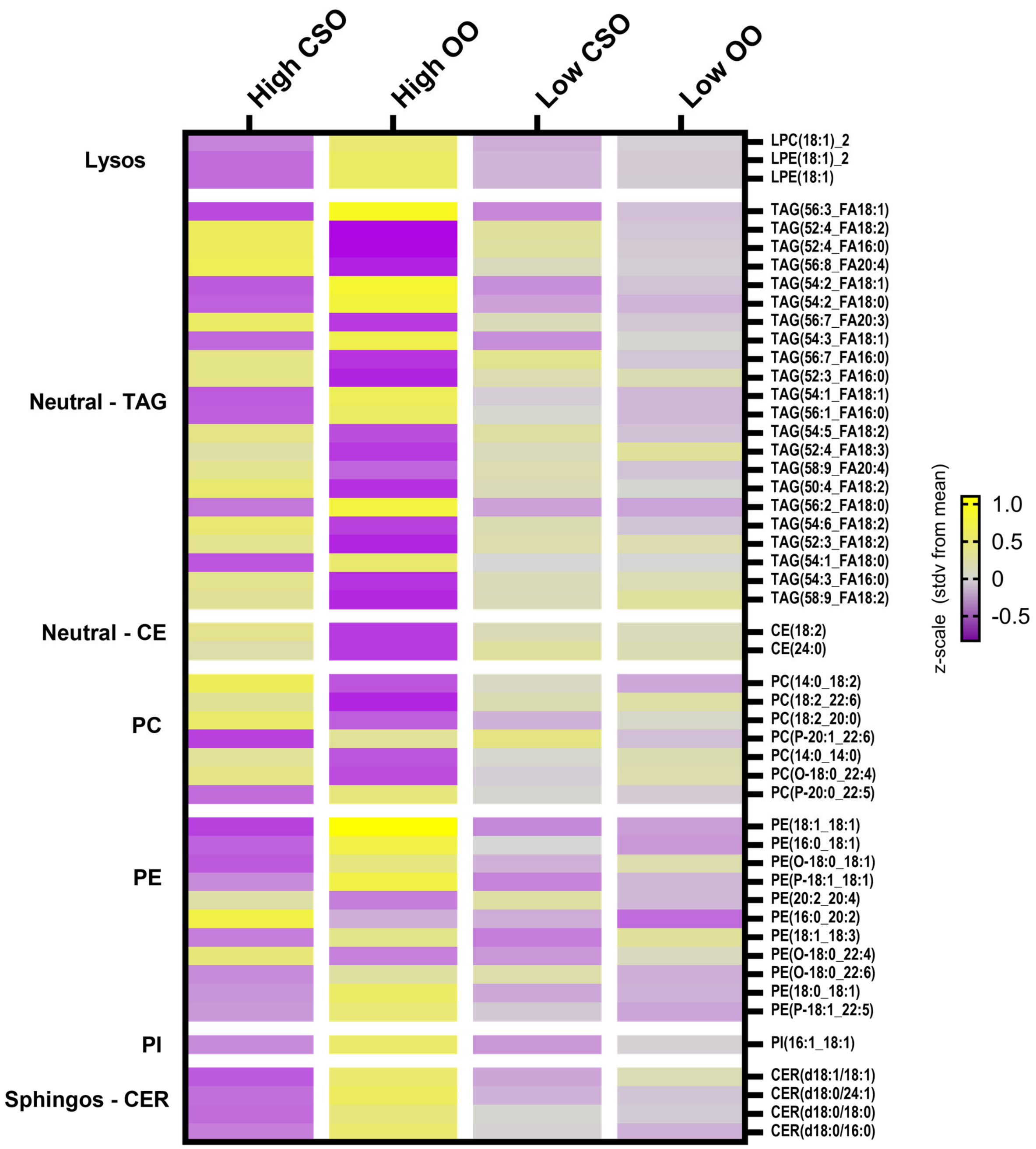

Figure S5: Heatmap of lipids that changed significantly between high CSO and OO grouped by functional class. Dietary groups are in columns and lipids are in rows, and their intersection represents the average intensity (z-score) relative to the average across all groups. Lipids displayed have $p<0.1$ via ANOVA. Purple indicates low abundance and yellow indicates high abundance. Lipids are grouped according to their acyl chain length.

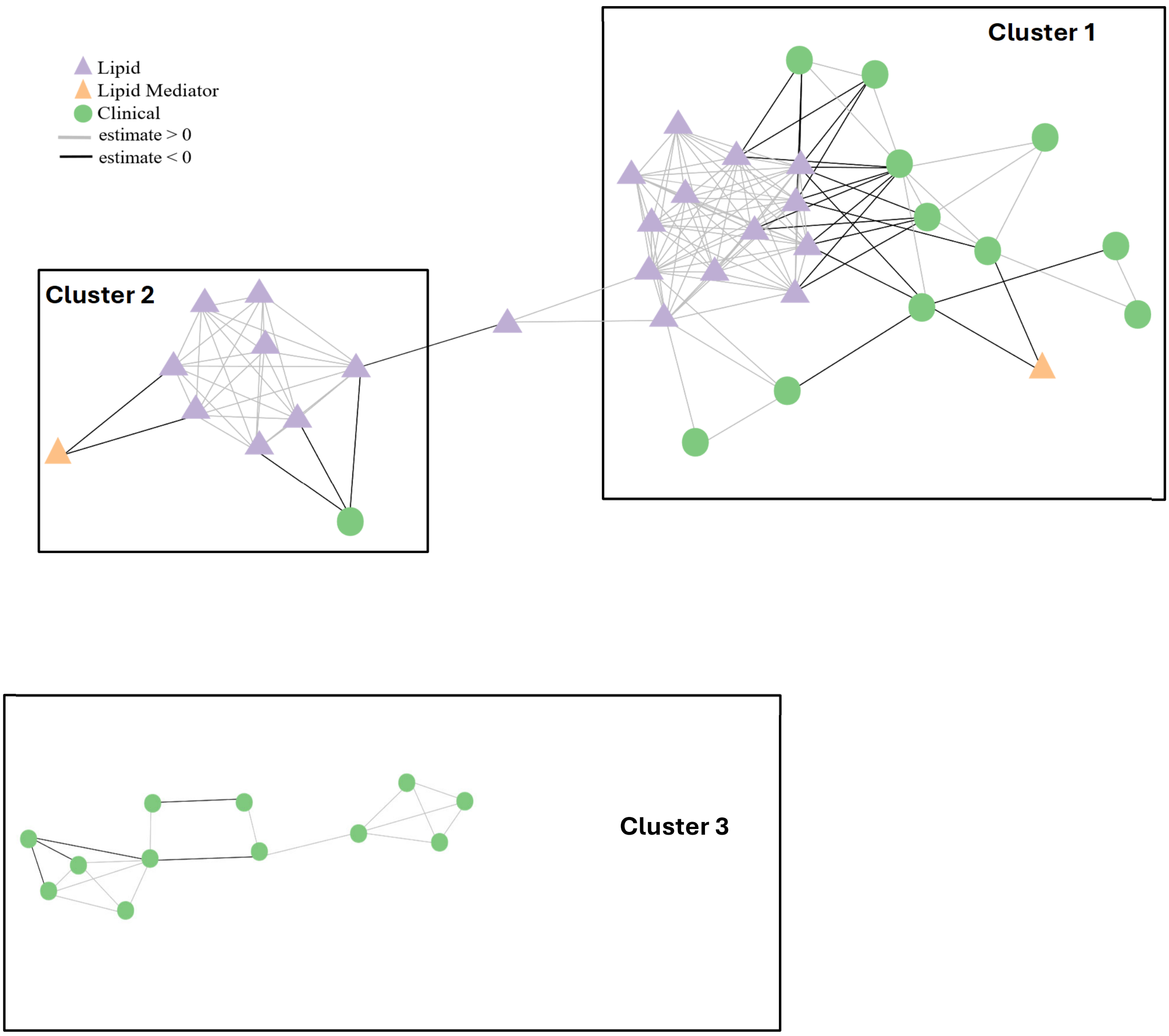

Figure S6: Network analysis of all statistically significant lipids, LMs and clinical covariates. Lipids (purple triangles) and LMs (orange triangles) that were determined as statistically significant from previous ANOVA analysis were combined with clinical covariates (green circles) to perform a network analysis. Gray edges represent positive correlations, and black edges represent negative correlations.

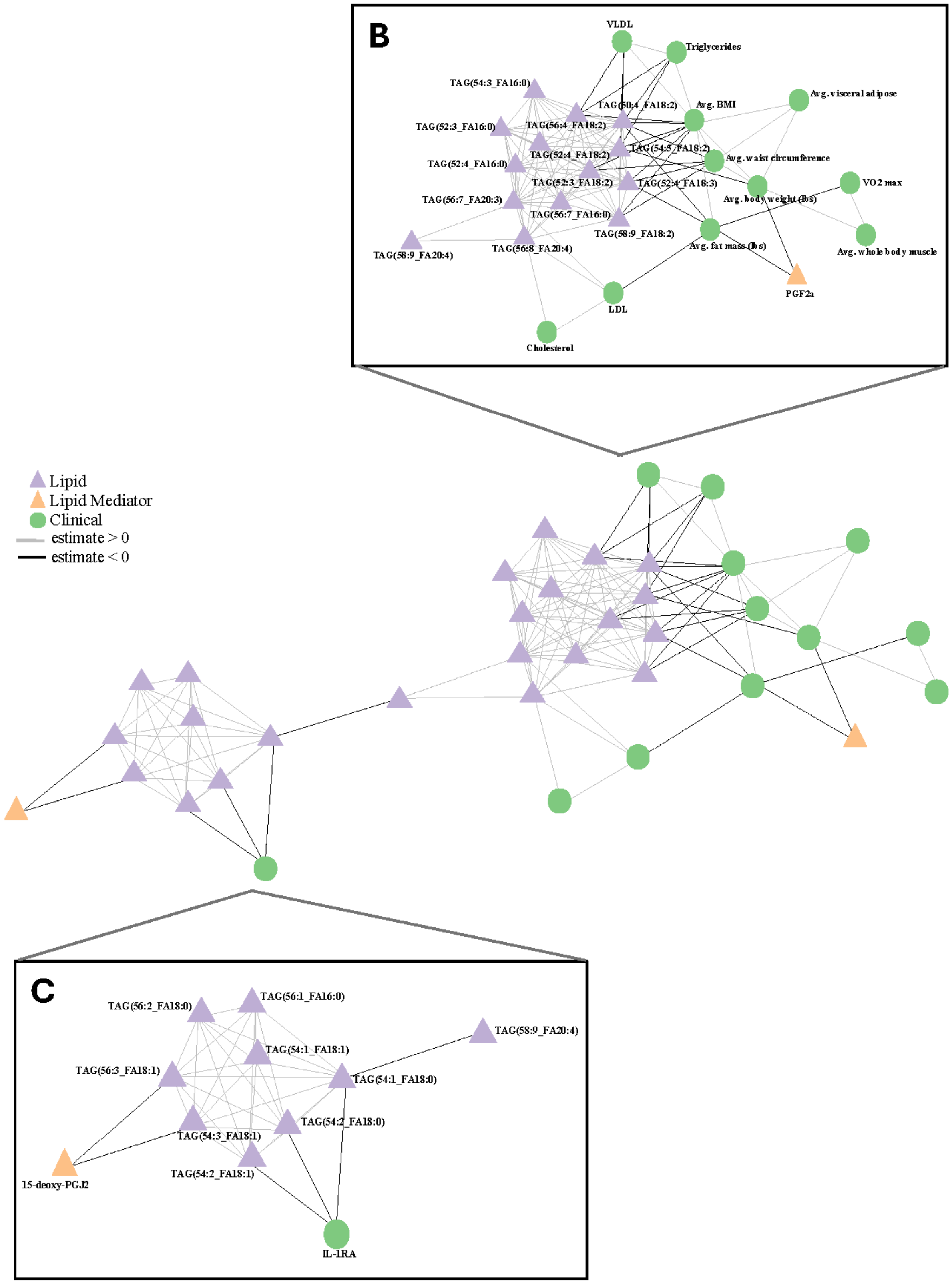
B
VLDL
Triglycerides
TAG(54:3_FA16:0)
TAG(50:4_FA18:2)
Avg. BMI
Avg. visceral adipose
TAG(52:3_FA16:0)
TAG(56:4_FA18:2)
TAG(52:4_FA18:2)
TAG(54:5_FA18:2)
Avg. waist circumference
TAG(52:4_FA16:0)
TAG(52:3_FA18:2)
TAG(52:4_FA18:3)
VO2 max
TAG(56:7_FA20:3)
Avg. body weight (lbs)
TAG(56:7_FA16:0)
TAG(58:9_FA18:2)
Avg. whole body muscle
TAG(58:9_FA20:4)
TAG(56:8_FA20:4)
Avg. fat mass (lbs)
PGF2a
LDL
Cholesterol
Lipid
Lipid Mediator
Clinical
estimate > 0
estimate < 0
C
TAG(56:2_FA18:0)
TAG(56:1_FA16:0)
TAG(58:9_FA20:4)
TAG(54:1_FA18:1)
TAG(56:3_FA18:1)
TAG(54:1_FA18:0)
TAG(54:2_FA18:0)
TAG(54:3_FA18:1)
TAG(54:2_FA18:1)
15-deoxy-PGJ2
IL-1RA

Figure S7: Zoomed in version of Figure 4.

Participant ID	Group	Additional oil consumed (g/day)
C01	60g OO	3.97
C02	60g CSO	1.16
C03	60g OO	0.43
C07	60g CSO	3.80
C11	30g OO	0.41
C12	60g CSO	5.75
C13	30g CSO	4.31
C14	30g CSO	1.23
C15	30g CSO	4.96
C16	30g OO	2.54
C17	30g CSO	1.10
C18	30g OO	23.52
C19	30g OO	0.64
C20	60g CSO	8.28
C21	60g CSO	4.46
C22	60g OO	4.56
C23	60g OO	0.42
C24	60g CSO	3.43
C26	30g CSO	0.28
C27	30g OO	8.38
C28	30g OO	0.26
C31	30g CSO	0.18
C32	60g OO	4.46
C33	30g OO	4.16
C35	60g CSO	1.94
C36	60g CSO	1.05
C37	30g CSO	1.88
C39	60g OO	2.76
C40	30g OO	0.56
C41	60g CSO	8.46
C42	30g CSO	0.57
C43	60g CSO	0.73
C44	60g OO	0.45
C45	30g CSO	0.57
C46	30g OO	3.52
C47	60g OO	1.29
C48	60g OO	1.85
C49	30g OO	6.42
C51	60g CSO	0.42
C53	60g OO	0.33
C54	60g CSO	3.48

C55	30g CSO	0.24
C56	60g OO	0.78
C59	30g OO	4.39
C60	30g OO	1.15
C62	30g CSO	4.16
C63	60g OO	4.6

Table S4: Amount of additional oil participants utilized for cooking purposes throughout the study.
